# Genomic sequencing of *Xanthomonas citri* pathovars that cause diseases in tropical fruit and cotton plants

**DOI:** 10.1590/1678-4685-GMB-2025-0079

**Published:** 2026-05-15

**Authors:** Lucas Pontes de Lucena, Juan Carlos Ariute, Ana Maria Benko-Iseppon, Bertram Brenig, Vasco Azevedo, Flávia Figueira Aburjaile, Keyla Walescka Lopes da Silva, André da Silva Xavier, Elineide Barbosa de Souza, Marco Aurélio Siqueira da Gama

**Affiliations:** 1 Universidade Federal Rural de Pernambuco (UFRPE), Departamento de Agronomia, Recife, PE, Brazil.; 2 Universidade Federal de Minas Gerais (UFMG), Escola de Veterinária, Departamento de Medicina Veterinária Preventiva, Belo Horizonte, MG, Brazil.; 3 Universidade Federal de Pernambuco (UFPE), Departamento de Genética, Recife, PE, Brazil.; 4 University Göttingen, Institute of Veterinary Medicine, Göttingen, Germany.; 5 Universidade Federal de Minas Gerais (UFMG), Instituto de Ciências Biológicas, Departamento de Genética, Ecologia e Evolução, Belo Horizonte, MG, Brazil.; 6 Universidade Federal Rural de Pernambuco (UFRPE), Departamento de Biologia, Recife, PE, Brazil.

**Keywords:** Plant pathogenic bacteria, *Citrus* spp., Mangiferae indicae, Vitis vinifera, *Gossypium* spp

## Abstract

*Xanthomonas citri* is a phytopathogenic species with a wide host range that harbors subspecies and pathovars such as *citri, mangiferaeindicae, viticola*, and *malvacearum* which cause citrus canker, bacterial canker of the mango, bacterial blight of cotton, and grapevine bacterial canker, respectively. In this work, 32 genomes belonging to these four pathovars were sequenced, two of which were from *X. citri* subsp. *citri*, one from *X. citri* pv. *mangiferaeindicae*, 13 from *X. citri* pv. *viticola*, and 16 from *X. citri* subsp. *malvacearum*. The genome size, N50, and GC content ranged from 4,978,419 to 5,256,019 bp, 67,322 to 727,867 bp, and 64.32% and 64.79%, respectively. All strains presented values of dDDH and ANIm greater than 77.3% and 96.1%, when compared to each other, and above 93% and 99%, when compared in relation to their respective pathotype strains. Maximum likelihood analysis of 1,298 core genes grouped the strains and their respective pathotype type with a 100% bootstrap. CRISPR sequences and genes from the Xop family (*XopX*, *XopA*, and *XopV*) and transcription activator-like effectors (TALE) were detected in all analyzed strains. The genomes sequenced in this study will help to understand the biology of these plant pathogens, enabling the development of more effective and precise management strategies and the prevention of disease outbreaks.

The species *Xanthomonas citri* has a wide range of hosts, is present on all inhabited continents, and stands out as one of the main biotic factors capable of limiting the economic return of several plant species (Shahbaz et al., 2022). Interestingly, strains within this species present specificity to different hosts and have been classified as pathovars and subspecies (Schaad et al., 2005; Ah-You et al., 2009).

Citrus canker is caused by *X. citri* subsp. *citri*, one of the main diseases of citrus crops and has been identified in several growing regions. The symptoms caused by the pathogen begin with small yellowish spots that become larger and protrude, giving the appearance of canker. These symptoms can be observed on leaves, branches, and fruits (Shahbaz et al., 2022). Mango bacterial canker is caused by *X. citri* pv. *mangiferaeindicae*. The main symptoms of the disease are necrotic leaf spots restricted by the veins, in some cases surrounded by a yellowish halo. The bacterium is also capable of infecting mango fruits, which under high severities, can produce infectious exudation, and cause premature fall (Gama et al., 2018). Cotton bacterial blight is caused by *X. citri* subsp. *malvacearum*. The disease’s initial symptoms are small, waterlogged leaf spots, which evolve into angular necrotic spots restricted by the veins. As the disease progresses, the bacterium can also cause lesions on the veins of the leaves. Lesions tend to darken, resulting in a burning appearance. Necroses can also be observed in cotton apples (Wang et al., 2019). In turn, grapevine bacterial canker is caused by *X. citri* pv. *viticola*, causing necrotic spots on the leaves, branches, veins, and petioles, which can evolve into fissures and cankers. Symptoms can be observed on the berries, which also show dark spots (Gama *et al*., 2018).

In the past, the *X. citri* subsp. *citri*, *X. citri* pv. *mangiferaeindicae, X. citri* pv. *viticola*, and *X. citri* subsp. *malvacearum*, were classified as *X. campestris* or *X. axonopodis* pathovars (Vauterin et al., 1995). However, over the years different studies based on DNA-DNA hybridization, multilocus sequence analyses of housekeeping genes, and genomic analyses reclassified these bacteria with *X. citri* (Schaad et al., 2005, Ah-You et al., 2009, Gama et al., 2018). However, although these pathovars share a high level of taxogenomic identity and phylogenomic relationship, the taxonomic status of these pathogens still needs to be better understood. In addition, many questions regarding the mechanisms involved in host range and pathogen-host interactions remain unresolved. Therefore, this study aimed to conduct genome sequencing of the strains of these pathovars deposited in the Rosa Mariano Culture Collection (RMCC) from the Phytobacteriology Laboratory (LAFIBAC) of Universidade Federal Rural de Pernambuco (UFRPE) to provide resources that may help to understand these aspects.

Over the years, strains of *X. citri* subsp. *citri* (2), *X. citri* pv. *mangiferaeindicae* (1)*, X. citri* pv. *viticola* (13), and *X. citri* subsp. *malvacearum* (16) isolated from leaves of their respective hosts have been characterized (Gama et al., 2011; Oliveira et al., 2011; Gama *et al*., 2018; Amancio et al., 2021) and deposited in the RMCC. These strains were cultivated in NYDA medium (20 g of agar, 10 g of dextrose, 5 g of peptone, 5 g of yeast extract, 3 g of meat extract, and one liter of sterilized distilled water), being subsequently incubated in a BOD-type chamber at 29 °C for 36 h. After this period, the isolated colonies were selected for DNA extraction using the MiniPrep bacterial DNA extraction kit (Axygen Biosciences, Tewksbury, MA) according to the manufacturer’s instructions. The libraries construction was carried out according to the manufacturer’s recommendations, and genome sequencing was carried out using the Illumina HiSeq 2500 platform (Illumina, San Diego, CA), constructing paired-end libraries with 150bp reads. The quality of the reads was then evaluated using FastQC software (Andrews, 2010). Adapters and low-quality reads were removed using Trimmomatic v. 0.32 (Bolger et al., 2014), and then the genomes were assembled de novo with the SPAdes (Bankevich et al., 2012). Assembly data were obtained using QUAST (Gurevich et al., 2013) and automatic annotation of the genomes was performed using Rapid Annotations using Subsystems Technology (Overbeek et al., 2013). The completeness of the assembled genomes was assessed using CheckM software, and the presence of plasmids was assessed using PlasmidFinder. The data obtained in these analyses were analyzed together with data from pathotype strains of the *X. citri* pathovars obtained from the GenBank database (https://www.ncbi.nlm.nih.gov/genbank/). The average nucleotide identity based on the Mummer aligner was calculated using pyani v.0.2.11 (Pritchard et al., 2016), DNA-DNA hybridization was performed using the GGDC platform (Meier-Kolthoff et al., 2021), and orthologous genes were obtained using the Roary pipeline (Page et al., 2015). Orthologous genes were aligned using Mafft 7.487 (Katoh and Standley, 2013), and a maximum likelihood tree was constructed using IQ-TREE (Trifinopoulos et al., 2016). Analyses of CRISPR sequences were carried out using CRISPRminer (Zhang et al., 2018). Effector proteins of the types III secretion systems were predicted using the programs T3SEPP (Hui et al., 2020) with the default parameters. The predicted proteins were evaluated using BLASTP from the GenBank database. 

The size, number of coding sequences, N50, and GC content of the *X. citri* genomes analyzed in this study ranged from 4,978,419 bp (subsp. *malvacearum,* strain CRMXCM242) to 5,256,019 bp (pv. *viticola*, strain CCRMXCV214), 4903 (pv. *viticola*, strain CCRMXCV13) to 4650 sequences (subsp. *malvacearum*, strain CRMXCM424.1), 727,867 bp (pv. *viticola*, strain CCRMXCV154) to 67,322 bp (pv. *mangiferaeindicae,* strain CCRM1M), and 64.79% (pv. *mangiferaeindicae,* strain CCRM1M) to 64.32% (pv. *viticola*, CCRMXCV13), respectively (Table 1). The contig size ranged from 1,527,958 bp (pv. *viticola,* CCRMXCV13) to 167,874 bp (pv. *mangiferaeindicae,* strain CCRM1M), while the quantity of RNAs ranged from 56 RNAs (pv. *citri* CCRM1Ci and CCRM6Ci) to 54 RNAs (pv. *mangiferaeindicae,* strain CCRM1M), respectively. No plasmids were detected in the genomes sequenced and all strains were 100% completed.

**Table 1 -:** Genome data of *Xanthomonas citri* pv. *citri*, *X. citri* pv. *mangiferaeindicae*, *X. citri* pv. *vitícola*, and *X. citri* pv. *malvacearum s*trains sequenced in this study.

Species	Strain	Genome length	GC content (%)	Number of coding sequences	Largest contig	N50	Number of RNAs	Number of contigs	Genome Acession
*Xanthomonas citri* pv. *citri*	CCRM1Ci	5197089	64.75	4821	527416	223493	56	71	JAUNYC000000000
	CCRM6Ci	5198055	64.76	4819	533654	223223	56	70	JAUNYD000000000
*Xanthomonas citri* pv. *mangiferaeindicae*	CCRM1M	5054542	64.79	4795	167874	67322	54	185	JAUNYB000000000
*Xanthomonas citri* pv. *viticola*	CCRMXCV13	5263262	64.32	4903	1527958	697019	55	43	JBTJJY000000000
	CCRMXCV26	5250586	64.36	4888	1527929	510992	55	46	JBTJJX000000000
	CCRMXCV40	5202584	64.37	4859	1527914	571934	55	44	JBTJJW000000000
	CCRMXCV54	5209027	64.33	4874	1527930	510973	55	43	JBTCBM000000000
	CCRMXCV65	5202798	64.37	4847	1527973	697017	55	43	JBTCBL000000000
	CCRMXCV78	5076560	64.48	4669	1527802	510972	55	40	JBTCBK000000000
	CCRMXCV116	5201090	64.37	4853	1418535	600665	55	45	JBTCBJ000000000
	CCRMXCV124	5202376	64.37	4841	1166299	379235	55	40	JBTCBI000000000
	CCRMXCV214	5256019	64.36	4888	1527844	697011	55	42	JAUNYX000000000
	CCRMXCV33	5201517	64.37	4838	1527746	696996	55	42	JAUNYU000000000
	CCRMXCV154	5255359	64.36	4883	1527888	727867	55	37	JAUNYY000000000
	CCRMXCV230	5074786	64.49	4683	1527848	696822	55	39	JAUNYW000000000
	CCRMXCV234	5077869	64.48	4679	1527857	727863	55	36	JAUNYV000000000
*Xanthomonas citri* pv. *malvacearum*	CRMXCM131	4980656	64.7	4655	357903	134798	55	113	JAUNYI000000000
	CRMXCM223	4980613	64.7	4651	357903	134797	55	114	JAUNYO000000000
	CRMXCM233	4980954	64.7	4662	357903	134797	55	112	JAUNYN000000000
	CRMXCM242	4978419	64.7	4668	343067	134797	55	113	JAUNYS000000000
	CRMXCM414	4980208	64.7	4660	299076	118427	55	117	JAUNYG000000000
	CRMXCM423	4980083	64.7	4662	357890	134797	55	113	JAUNYL000000000
	CRMXCM424.1	4980246	64.7	4650	357890	134797	55	113	JAUNYT000000000
	CRMXCM424.2	4980238	64.7	4652	357890	134797	55	113	JAUNYH000000000
	CRMXCM431	4980218	64.7	4655	357890	134797	55	115	JAUNYE000000000
	CRMXCM432	4980767	64.7	4655	357890	134797	55	110	JAUNYP000000000
	CRMXCM441	4980237	64.7	4662	357890	134795	55	113	JAUNYJ000000000
	CRMXCM811	4979176	64.7	4654	357890	134797	55	113	JAUNYQ000000000
	CRMXCM1012	4978593	64.7	4671	343067	134797	55	113	JAUNYR000000000
	CRMXCM1033	4979964	64.7	4658	357903	134797	55	113	JAUNYM000000000
	CRMXCM1133	4980110	64.7	4667	343073	134797	55	114	JAUNYK000000000
	CRMXCM4021	5013614	64.68	4696	357896	134797	55	115	JAUNYF000000000

The ANI analyses demonstrated values above 96.1% when comparing all the strains analyzed, and the values observed in the DDH analyses were all higher than 77.3% (Figure 1 A ). When comparing the strains sequenced in this study with their respective pathovars, the values of ANI and dDDH were 99.9% and 99.2% for *X*. *citri* pv. *citri*, 99.9% and 97.2% for *X. citri* pv. *mangiferaeindicae*, 99.9% and above 99% for *X. citri* pv. *viticola*, and above 99.5% and 93.6% for *X. citri* subsp. *malvacearum*. Thus, considering all strains evaluated showed ANI and dDDH values concerning type strain of *X. citri* (LMG9322^T^) greater than 95% and 70%, which are the standard values for bacterial species differentiation (Gama et al., 2018), it was confirmed that the strains sequenced in this work belong to the *X. citri*. Regarding orthologous genes, out of a total of 13,647 genes, 1,298 core genes, 1,691 soft core genes, 2,886 shell genes, and 7,772 cloud genes were detected. Maximum likelihood tree analysis performed with core genes divided the *X. citri* strains into two phylogenetic groups, as observed in previous studies (Gama *et al*., 2018; Lucena et al., 2023), and the strains sequenced in this study were grouped with the pathotype strains of their respective pathovars with a 100% bootstrap (Figure 1 B ).

**Figure 1 -:**
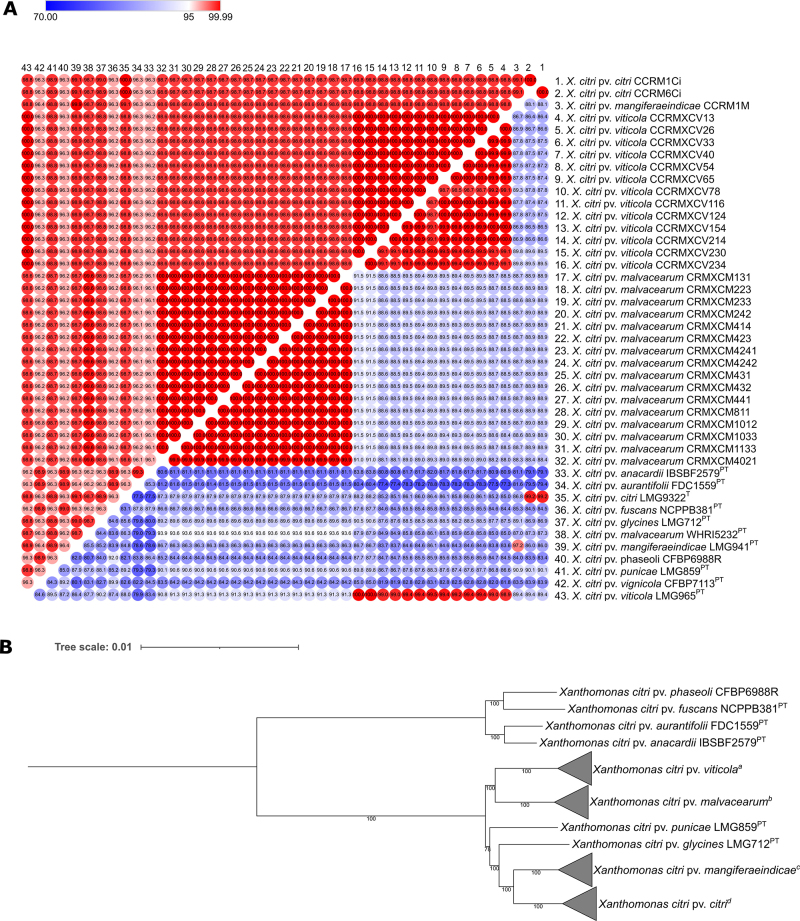
Taxogenomic and phylogenomic relationship of *Xanthomonas citri* subsp. *citri*, *X. citri* pv. *mangiferaeindicae*, *X. citri* pv. *viticola*, and *X. citri* subsp. *malvacearum*. (A) Heatmap representing the percentage of identity (average nucleotide identity based on the Mummer aligner [ANIm]) and digital DNA-DNA hybridization (dDDH) between pathovars of *X. citri*. The upper triangle shows the ANIm values, and the lower triangle shows the dDDH values. (B) Maximum-likelihood phylogenetic tree based on core genes of pathovars and subspecies of *X. citri*. Numbers at nodes are bootstrap values (>50%) from 1,000 repetitions. The bar represents the expected number of substitutions per site. Superscripts following strain names: T=type strain of a species, and PT=pathotype strain of a pathovar.

CRISPR sequences were detected in all analyzed strains. In addition, *cas* proteins information are presented in Figure 2. The presence of the cas3-type protein observed in strains of *X. citri* subsp. *citri* CCRM1Ci and CCRM6Ci indicate that both belong to CRISPR-cas locus type I (Makarova et al., 2015). DEDDh and Ding were the main domains associated with CRISPR. Among the strains evaluated, no domain related to CRISPR was found only in strains CCRMXCV124, CCRMXCV154, CCRMXCV214 and CCRMXCV230 of *X. citri* pv. *viticola*.

**Figure 2 -:**
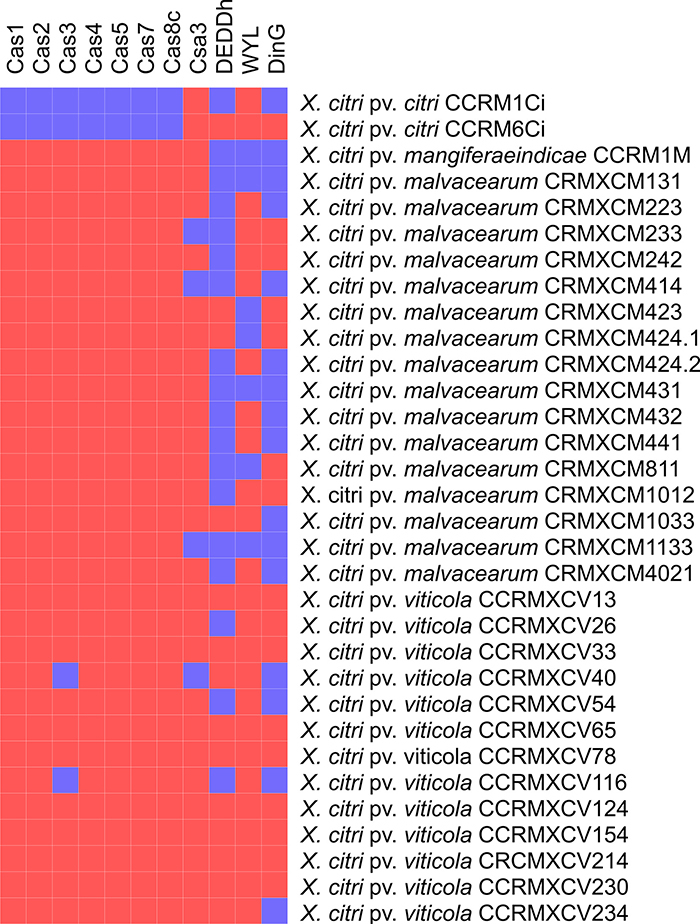
CRISPR data of *Xanthomonas citri* subsp. *citri*, *X. citri* pv. *mangiferaeindicae*, *X. citri* pv. *viticola*, and *X. citri* subsp. *malvacearum* strains sequenced in this study. Blue box indicates the presence of cas proteins, while the red box indicates the absence of cas proteins.

The genes predicted to be related to the type III secretion system demonstrated the presence of genes from the Xop family (*XopX*, *XopA*, and *XopV*) and from transcription activator-like effectors (TALE) (Table S1), in accordance with previous studies performed with different subspecies and pathovars of *X. citri* (White et al., 2009; Lucena et al., 2023). These genes vary at the interspecies level, and distinct sets of type III effectors have been identified in these different bacteria. Therefore, the functional investigation of these genes may shed light on the pathogenicity aspects and/or host specificity of the strains that compose this species (Okoh et al., 2025). 

The data available here provides resources capable of contributing to the knowledge of biology, ecology, genomics, and pathogenesis of the X. *citri* pvs. *citri*, *mangiferaeindicae*, *viticola*, and *malvacearum*, ensuring useful information for developing management strategies for diseases caused by these plant pathogenic bacteria.

## Data Availability

 The genome sequences generated in this study are available in GenBank under the accession numbers listed in Table 1.

## Supplementary material

The following online material is available for this article:

Table S1 - Type III effector proteins predicted in the genomes of the *Xanthomonas citri* subsp. *citri*, *X. citri* pv*. mangiferaeindicae*, *X. citri* pv. *viticola*, and *X. citri* subsp*. malvacearum* strains sequenced in this study.
