# Supplementary material for: Genomic sequencing of Xanthomonas citri pathovars that cause diseases in tropical fruit and cotton plants
Source: Genet Mol Biol. 2026 May 15;49(1):e20250079. doi: 10.1590/1678-4685-GMB-2025-0079 (PMC13178084; doi:10.1590/1678-4685-GMB-2025-0079)
Supplement: Table S1 - [file 1415-4757-GMB-49-1-e20250079-s1.pdf]

Genetics and Molecular Biology
https://doi.org/10.1590/1678-4685-GMB-2025-0079

Supplementary Material to "Genomic sequencing of *Xanthomonas citri* pathovars that cause diseases in tropical fruit and cotton plants"

Table S1 - Type III effector proteins predicted in the genomes of the *Xanthomonas citri* subsp. *citri*, *X. citri* pv. *mangiferaeindicae*, *X. citri* pv. *viticola*, and *X. citri* subsp. *malvacearum* strains sequenced in this study.

Xanthomonas citri pv. *citri* CCRM1Ci		
Protein	Prediction	Description
fig|6666666.1076482.peg.3350	T3S	head completion/stabilization protein
fig|6666666.1076482.peg.1888	T3S	XopX family type III secretion system effector
fig|6666666.1076482.peg.1015	T3S	4-hydroxyphenylacetate catabolism regulator HpaA
fig|6666666.1076482.peg.1022	T3S	HrpF/NolX family T3SS translocon protein
fig|6666666.1076482.peg.1077	T3S	cellulase family glycosylhydrolase
fig|6666666.1076482.peg.1084	T3S	Hypothetical Protein
fig|6666666.1076482.peg.1094	T3S	type III secretion system effector avirulence protein AvrBs2
fig|6666666.1076482.peg.1177	T3S	CPBP family intramembrane glutamic endopeptidase
fig|6666666.1076482.peg.1244	T3S	TonB-like protein
fig|6666666.1076482.peg.1344	T3S	L-fucose:H+ symporter permease
fig|6666666.1076482.peg.1404	T3S	Crp/Fnr family transcriptional regulator
X. citri pv. *citri* CCRM6Ci		
Protein	Prediction	Description
fig|6666666.1076486.peg.3221	T3S	head completion/stabilization protein
fig|6666666.1076486.peg.1551	T3S	XopX family type III secretion system effector
fig|6666666.1076486.peg.1030	T3S	L-fucose:H+ symporter permease
fig|6666666.1076486.peg.1130	T3S	TonB-like protein
fig|6666666.1076486.peg.1149	T3S	Crp/Fnr family transcriptional regulator
fig|6666666.1076486.peg.1222	T3S	hypothetical protein
fig|6666666.1076486.peg.1226	T3S	methyl-accepting chemotaxis protein
fig|6666666.1076486.peg.1256	T3S	hypothetical protein
fig|6666666.1076486.peg.1257	T3S	SymE family type I addiction module toxin
fig|6666666.1076486.peg.1315	T3S	hypothetical protein
X. citri pv. *mangiferaeindicae* CCRM1M		
Protein	Prediction	Description
fig|6666666.1076488.peg.4734	T3S	head completion/stabilization protein
fig|6666666.1076488.peg.539	T3S	hypothetical protein
fig|6666666.1076488.peg.1100	T3S	protein adenylyltransferase SelO family protein
fig|6666666.1076488.peg.1114	T3S	Crp/Fnr family transcriptional regulator
fig|6666666.1076488.peg.1182	T3S	Avirulence protein AvrBs3

fig|6666666.1076488.peg.1198	T3S	cellulase family glycosylhydrolase
fig|6666666.1076488.peg.1252	T3S	HrpF/NolX family T3SS translocon protein
fig|6666666.1076488.peg.1260	T3S	hypothetical protein
fig|6666666.1076488.peg.1276	T3S	elicitor of hypersensitive response HpaG
fig|6666666.1076488.peg.1277	T3S	hypothetical protein
X. citri pv. *malvacearum* CRMXCM131		
Protein	Prediction	Description
fig|6666666.1076504.peg.1004	T3S	hypothetical protein
fig|6666666.1076504.peg.1069	T3S	L-fucose:H+ symporter permease
fig|6666666.1076504.peg.1227	T3S	glycosyltransferase
fig|6666666.1076504.peg.1228	T3S	GumJ protein
fig|6666666.1076504.peg.1269	T3S	hypothetical protein
fig|6666666.1076504.peg.1396	T3S	FG-GAP repeat protein
fig|6666666.1076504.peg.1398	T3S	hypothetical protein
fig|6666666.1076504.peg.1399	T3S	VirB4 family type IV secretion/conjugal transfer ATPase
fig|6666666.1076504.peg.1404	T3S	hypothetical protein
fig|6666666.1076504.peg.1444	T3S	NADH-quinone oxidoreductase subunit NuoF
X. citri pv. *malvacearum* CRMXCM223		
Protein	Prediction	Description
fig|6666666.1076509.peg.1010	T3S	hypothetical protein
fig|6666666.1076509.peg.1075	T3S	L-fucose:H+ symporter permease
fig|6666666.1076509.peg.1223	T3S	hypothetical protein
fig|6666666.1076509.peg.1265	T3S	GumJ protein
fig|6666666.1076509.peg.1266	T3S	glycosyltransferase
fig|6666666.1076509.peg.1401	T3S	FG-GAP repeat protein
fig|6666666.1076509.peg.1403	T3S	hypothetical protein
fig|6666666.1076509.peg.1404	T3S	VirB4 family type IV secretion/conjugal transfer ATPase
fig|6666666.1076509.peg.1409	T3S	hypothetical protein
fig|6666666.1076509.peg.1449	T3S	NADH-quinone oxidoreductase subunit NuoF
X. citri pv. *malvacearum* CRMXCM233		
Protein	Prediction	Description
fig|6666666.1076510.peg.3841	T3S	head completion/stabilization protein
fig|6666666.1076510.peg.1075	T3S	XopX family type III secretion system effector
fig|6666666.1076510.peg.1098	T3S	DUF3426 domain-containing protein
fig|6666666.1076510.peg.1118	T3S	CD225/dispanin family protein
fig|6666666.1076510.peg.1146	T3S	hypothetical protein
fig|6666666.1076510.peg.1200	T3S	L-fucose:H+ symporter permease
fig|6666666.1076510.peg.1369	T3S	glycosyltransferase
fig|6666666.1076510.peg.1370	T3S	GumJ protein
fig|6666666.1076510.peg.1411	T3S	hypothetical protein
fig|6666666.1076510.peg.1456	T3S	TrmH family RNA methyltransferase
X. citri pv. *malvacearum* CRMXCM242		

Protein	Prediction	Description
fig|6666666.1076518.peg.1000	T3S	hypothetical protein
fig|6666666.1076518.peg.1054	T3S	L-fucose:H+ symporter permease
fig|6666666.1076518.peg.1213	T3S	hypothetical protein
fig|6666666.1076518.peg.1255	T3S	GumJ protein
fig|6666666.1076518.peg.1256	T3S	glycosyltransferase
fig|6666666.1076518.peg.1433	T3S	NADH-quinone oxidoreductase subunit NuoF
fig|6666666.1076518.peg.1473	T3S	hypothetical protein
fig|6666666.1076518.peg.1478	T3S	VirB4 family type IV secretion/conjugal transfer ATPase
fig|6666666.1076518.peg.1479	T3S	hypothetical protein
fig|6666666.1076518.peg.1481	T3S	FG-GAP repeat protein
X. citri pv. *malvacearum* CRMXCM414		
Protein	Prediction	Description
fig|6666666.1076517.peg.3861	T3S	head completion/stabilization protein
fig|6666666.1076517.peg.885	T3S	XopX family type III secretion system effector
fig|6666666.1076517.peg.1010	T3S	L-fucose:H+ symporter permease
fig|6666666.1076517.peg.1170	T3S	hypothetical protein
fig|6666666.1076517.peg.1185	T3S	hypothetical protein
fig|6666666.1076517.peg.1212	T3S	GumJ protein
fig|6666666.1076517.peg.1213	T3S	glycosyltransferase
fig|6666666.1076517.peg.1348	T3S	FG-GAP repeat protein
fig|6666666.1076517.peg.1350	T3S	hypothetical protein
fig|6666666.1076517.peg.1351	T3S	VirB4 family type IV secretion/conjugal transfer ATPase
X. citri pv. *malvacearum* CRMXCM423		
Protein	Prediction	Description
fig|6666666.1076524.peg.1074	T3S	L-fucose:H+ symporter permease
fig|6666666.1076524.peg.1232	T3S	glycosyltransferase
fig|6666666.1076524.peg.1233	T3S	GumJ protein
fig|6666666.1076524.peg.1274	T3S	hypothetical protein
fig|6666666.1076524.peg.1401	T3S	FG-GAP repeat protein
fig|6666666.1076524.peg.1403	T3S	hypothetical protein
fig|6666666.1076524.peg.1404	T3S	VirB4 family type IV secretion/conjugal transfer ATPase
fig|6666666.1076524.peg.1409	T3S	hypothetical protein
fig|6666666.1076524.peg.1449	T3S	NADH-quinone oxidoreductase subunit NuoF
fig|6666666.1076524.peg.1667	T3S	class III extradiol ring-cleavage dioxygenase
X. citri pv. *malvacearum* CRMXCM4241		
Protein	Prediction	Description
fig|6666666.1076523.peg.1005	T3S	hypothetical protein
fig|6666666.1076523.peg.1070	T3S	L-fucose:H+ symporter permease
fig|6666666.1076523.peg.1218	T3S	hypothetical protein
fig|6666666.1076523.peg.1260	T3S	GumJ protein
fig|6666666.1076523.peg.1261	T3S	glycosyltransferase

fig|6666666.1076523.peg.1438	T3S	NADH-quinone oxidoreductase subunit NuoF
fig|6666666.1076523.peg.1478	T3S	hypothetical protein
fig|6666666.1076523.peg.1483	T3S	VirB4 family type IV secretion/conjugal transfer ATPase
fig|6666666.1076523.peg.1484	T3S	hypothetical protein
fig|6666666.1076523.peg.1486	T3S	FG-GAP repeat protein
X. citri pv. *malvacearum* CRMXCM4242		
Protein	Prediction	Description
fig|6666666.1076533.peg.3863	T3S	head completion/stabilization protein
fig|6666666.1076533.peg.935	T3S	XopX family type III secretion system effector
fig|6666666.1076533.peg.1006	T3S	hypothetical protein
fig|6666666.1076533.peg.1060	T3S	L-fucose:H+ symporter permease
fig|6666666.1076533.peg.1228	T3S	glycosyltransferase
fig|6666666.1076533.peg.1229	T3S	GumJ protein
fig|6666666.1076533.peg.1270	T3S	hypothetical protein
fig|6666666.1076533.peg.1397	T3S	FG-GAP repeat protein
fig|6666666.1076533.peg.1399	T3S	hypothetical protein
fig|6666666.1076533.peg.1400	T3S	VirB4 family type IV secretion/conjugal transfer ATPase
X. citri pv. *malvacearum* CRMXCM431		
Protein	Prediction	Description
fig|6666666.1076534.peg.1012	T3S	hypothetical protein
fig|6666666.1076534.peg.1076	T3S	L-fucose:H+ symporter permease
fig|6666666.1076534.peg.1234	T3S	glycosyltransferase
fig|6666666.1076534.peg.1235	T3S	GumJ protein
fig|6666666.1076534.peg.1276	T3S	hypothetical protein
fig|6666666.1076534.peg.1441	T3S	NADH-quinone oxidoreductase subunit NuoF
fig|6666666.1076534.peg.1481	T3S	hypothetical protein
fig|6666666.1076534.peg.1486	T3S	VirB4 family type IV secretion/conjugal transfer ATPase
fig|6666666.1076534.peg.1487	T3S	hypothetical protein
fig|6666666.1076534.peg.1489	T3S	FG-GAP repeat protein
X. citri pv. *malvacearum* CRMXCM432		
Protein	Prediction	Description
fig|6666666.1076535.peg.1012	T3S	hypothetical protein
fig|6666666.1076535.peg.1096	T3S	DUF3426 domain-containing protein
fig|6666666.1076535.peg.1116	T3S	CD225/dispanin family protein
fig|6666666.1076535.peg.1144	T3S	hypothetical protein
fig|6666666.1076535.peg.1198	T3S	L-fucose:H+ symporter permease
fig|6666666.1076535.peg.1366	T3S	glycosyltransferase
fig|6666666.1076535.peg.1367	T3S	GumJ protein
fig|6666666.1076535.peg.1409	T3S	hypothetical protein
fig|6666666.1076535.peg.1472	T3S	hypothetical protein
fig|6666666.1076535.peg.1477	T3S	TrmH family RNA methyltransferase
X. citri pv. *malvacearum* CRMXCM441		

Protein	Prediction	Description
fig|6666666.1076537.peg.1074	T3S	L-fucose:H+ symporter permease
fig|6666666.1076537.peg.1222	T3S	hypothetical protein
fig|6666666.1076537.peg.1263	T3S	GumJ protein
fig|6666666.1076537.peg.1264	T3S	glycosyltransferase
fig|6666666.1076537.peg.1401	T3S	FG-GAP repeat protein
fig|6666666.1076537.peg.1403	T3S	hypothetical protein
fig|6666666.1076537.peg.1404	T3S	VirB4 family type IV secretion/conjugal transfer ATPase
fig|6666666.1076537.peg.1409	T3S	hypothetical protein
fig|6666666.1076537.peg.1449	T3S	NADH-quinone oxidoreductase subunit NuoF
fig|6666666.1076537.peg.1667	T3S	class III extradiol ring-cleavage dioxygenase
X. citri pv. *malvacearum* CRMXCM811		
Protein	Prediction	Description
fig|6666666.1076538.peg.1008	T3S	hypothetical protein
fig|6666666.1076538.peg.1062	T3S	L-fucose:H+ symporter permease
fig|6666666.1076538.peg.1231	T3S	glycosyltransferase
fig|6666666.1076538.peg.1232	T3S	GumJ protein
fig|6666666.1076538.peg.1273	T3S	hypothetical protein
fig|6666666.1076538.peg.1438	T3S	NADH-quinone oxidoreductase subunit NuoF
fig|6666666.1076538.peg.1478	T3S	hypothetical protein
fig|6666666.1076538.peg.1483	T3S	VirB4 family type IV secretion/conjugal transfer ATPase
fig|6666666.1076538.peg.1484	T3S	hypothetical protein
fig|6666666.1076538.peg.1486	T3S	FG-GAP repeat protein
X. citri pv. *malvacearum* CRMXCM1012		
Protein	Prediction	Description
fig|6666666.1076539.peg.1062	T3S	L-fucose:H+ symporter permease
fig|6666666.1076539.peg.1221	T3S	glycosyltransferase
fig|6666666.1076539.peg.1222	T3S	GumJ protein
fig|6666666.1076539.peg.1264	T3S	hypothetical protein
fig|6666666.1076539.peg.1391	T3S	FG-GAP repeat protein
fig|6666666.1076539.peg.1393	T3S	hypothetical protein
fig|6666666.1076539.peg.1394	T3S	VirB4 family type IV secretion/conjugal transfer ATPase
fig|6666666.1076539.peg.1399	T3S	hypothetical protein
fig|6666666.1076539.peg.1439	T3S	NADH-quinone oxidoreductase subunit NuoF
fig|6666666.1076539.peg.1589	T3S	hypothetical protein
X. citri pv. *malvacearum* CRMXCM1033		
Protein	Prediction	Description
fig|6666666.1076540.peg.1009	T3S	hypothetical protein
fig|6666666.1076540.peg.1074	T3S	L-fucose:H+ symporter permease
fig|6666666.1076540.peg.1232	T3S	glycosyltransferase
fig|6666666.1076540.peg.1233	T3S	GumJ protein

fig|6666666.1076540.peg.1274	T3S	hypothetical protein
fig|6666666.1076540.peg.1400	T3S	FG-GAP repeat protein
fig|6666666.1076540.peg.1402	T3S	hypothetical protein
fig|6666666.1076540.peg.1403	T3S	VirB4 family type IV secretion/conjugal transfer ATPase
fig|6666666.1076540.peg.1408	T3S	hypothetical protein
fig|6666666.1076540.peg.1448	T3S	NADH-quinone oxidoreductase subunit NuoF
X. citri pv. *malvacearum* CRMXCM1133		
Protein	Prediction	Description
fig|6666666.1076541.peg.1050	T3S	L-fucose:H+ symporter permease
fig|6666666.1076541.peg.1209	T3S	hypothetical protein
fig|6666666.1076541.peg.1251	T3S	GumJ protein
fig|6666666.1076541.peg.1252	T3S	glycosyltransferase
fig|6666666.1076541.peg.1389	T3S	FG-GAP repeat protein
fig|6666666.1076541.peg.1391	T3S	hypothetical protein
fig|6666666.1076541.peg.1392	T3S	VirB4 family type IV secretion/conjugal transfer ATPase
fig|6666666.1076541.peg.1397	T3S	hypothetical protein
fig|6666666.1076541.peg.1437	T3S	NADH-quinone oxidoreductase subunit NuoF
fig|6666666.1076541.peg.1586	T3S	hypothetical protein
X. citri pv. *malvacearum* CRMXCM4021		
Protein	Prediction	Description
fig|6666666.1076542.peg.1010	T3S	hypothetical protein
fig|6666666.1076542.peg.1065	T3S	L-fucose:H+ symporter permease
fig|6666666.1076542.peg.1234	T3S	glycosyltransferase
fig|6666666.1076542.peg.1235	T3S	GumJ protein
fig|6666666.1076542.peg.1275	T3S	hypothetical protein
fig|6666666.1076542.peg.1399	T3S	FG-GAP repeat protein
fig|6666666.1076542.peg.1401	T3S	hypothetical protein
fig|6666666.1076542.peg.1402	T3S	VirB4 family type IV secretion/conjugal transfer ATPase
fig|6666666.1076542.peg.1407	T3S	hypothetical protein
fig|6666666.1076542.peg.1447	T3S	NADH-quinone oxidoreductase subunit NuoF
X. citri pv. *viticola* CCRM13		
Protein	Prediction	Description
fig|6666666.1078948.peg.1923	T3S	peptidoglycan-binding protein
fig|6666666.1078948.peg.1119	T3S	cation diffusion facilitator family transporter
fig|6666666.1078948.peg.1215	T3S	efflux RND transporter periplasmic adaptor subunit
fig|6666666.1078948.peg.1216	T3S	DHA2 family efflux MFS transporter permease subunit
fig|6666666.1078948.peg.1221	T3S	hypothetical protein
fig|6666666.1078948.peg.1225	T3S	M3 family metallopeptidase
fig|6666666.1078948.peg.125	T3S	oligopeptide:H+ symporter
fig|6666666.1078948.peg.129	T3S	4-hydroxyphenylpyruvate dioxygenase
fig|6666666.1078948.peg.1339	T3S	L-fucose:H+ symporter permease

fig|6666666.1078948.peg.1442	T3S	TonB-like protein
X. citri pv. *viticola* CCRM26		
Protein	Prediction	Description
fig|6666666.1078949.peg.3290	T3S	peptidoglycan-binding protein
fig|6666666.1078949.peg.1120	T3S	cation diffusion facilitator family transporter
fig|6666666.1078949.peg.1217	T3S	efflux RND transporter periplasmic adaptor subunit
fig|6666666.1078949.peg.1218	T3S	DHA2 family efflux MFS transporter permease subunit
fig|6666666.1078949.peg.1223	T3S	hypothetical protein
fig|6666666.1078949.peg.1227	T3S	M3 family metallopeptidase
fig|6666666.1078949.peg.125	T3S	oligopeptide:H+ symporter
fig|6666666.1078949.peg.129	T3S	4-hydroxyphenylpyruvate dioxygenase
fig|6666666.1078949.peg.1341	T3S	L-fucose:H+ symporter permease
fig|6666666.1078949.peg.1444	T3S	TonB-like protein
X. citri pv. *viticola* CCRM33		
Protein	Prediction	Description
fig|6666666.1076496.peg.2430	T3S	peptidoglycan-binding protein
fig|6666666.1076496.peg.119	T3S	L-fucose:H+ symporter permease
fig|6666666.1076496.peg.1246	T3S	DUF3426 domain-containing protein
fig|6666666.1076496.peg.1264	T3S	CD225/dispanin family protein
fig|6666666.1076496.peg.1327	T3S	4-hydroxyphenylpyruvate dioxygenase
fig|6666666.1076496.peg.1331	T3S	oligopeptide:H+ symporter
fig|6666666.1076496.peg.1370	T3S	XopA/Hpa1 family type III secretion system protein
fig|6666666.1076496.peg.1379	T3S	type III secretion protein HrpB2
fig|6666666.1076496.peg.1387	T3S	4-hydroxyphenylacetate catabolism regulator HpaA
fig|6666666.1076496.peg.1392	T3S	hypothetical protein
X. citri pv. *viticola* CCRM40		
Protein	Prediction	Description
fig|6666666.1078951.peg.2737	T3S	peptidoglycan-binding protein
fig|6666666.1078951.peg.118	T3S	L-fucose:H+ symporter permease
fig|6666666.1078951.peg.1244	T3S	DUF3426 domain-containing protein
fig|6666666.1078951.peg.1262	T3S	CD225/dispanin family protein
fig|6666666.1078951.peg.1325	T3S	4-hydroxyphenylpyruvate dioxygenase
fig|6666666.1078951.peg.1329	T3S	oligopeptide:H+ symporter
fig|6666666.1078951.peg.1368	T3S	XopA/Hpa1 family type III secretion system protein
fig|6666666.1078951.peg.1377	T3S	type III secretion protein HrpB2
fig|6666666.1078951.peg.1385	T3S	4-hydroxyphenylacetate catabolism regulator HpaA
fig|6666666.1078951.peg.1390	T3S	hypothetical protein
X. citri pv. *viticola* CCRM54		
Protein	Prediction	Description
fig|6666666.1078952.peg.3274	T3S	peptidoglycan-binding protein
fig|6666666.1078952.peg.1012	T3S	hypothetical protein
fig|6666666.1078952.peg.1133	T3S	cation diffusion facilitator family transporter
fig|6666666.1078952.peg.1175	T3S	hypothetical protein

fig|6666666.1078952.peg.1230	T3S	efflux RND transporter periplasmic adaptor subunit
fig|6666666.1078952.peg.1231	T3S	DHA2 family efflux MFS transporter permease subunit
fig|6666666.1078952.peg.1236	T3S	hypothetical protein
fig|6666666.1078952.peg.1240	T3S	M3 family metallopeptidase
fig|6666666.1078952.peg.127	T3S	oligopeptide:H+ symporter
fig|6666666.1078952.peg.131	T3S	4-hydroxyphenylpyruvate dioxygenase
X. citri pv. *viticola* CCRM65		
Protein	Prediction	Description
fig|6666666.1078953.peg.1925	T3S	peptidoglycan-binding protein
fig|6666666.1078953.peg.1121	T3S	cation diffusion facilitator family transporter
fig|6666666.1078953.peg.1218	T3S	efflux RND transporter periplasmic adaptor subunit
fig|6666666.1078953.peg.1219	T3S	DHA2 family efflux MFS transporter permease subunit
fig|6666666.1078953.peg.1224	T3S	hypothetical protein
fig|6666666.1078953.peg.1228	T3S	M3 family metallopeptidase
fig|6666666.1078953.peg.124	T3S	oligopeptide:H+ symporter
fig|6666666.1078953.peg.128	T3S	4-hydroxyphenylpyruvate dioxygenase
fig|6666666.1078953.peg.1342	T3S	L-fucose:H+ symporter permease
fig|6666666.1078953.peg.1446	T3S	TonB-like protein
X. citri pv. *viticola* CCRM78		
Protein	Prediction	Description
fig|6666666.1078954.peg.3080	T3S	peptidoglycan-binding protein
fig|6666666.1078954.peg.116	T3S	L-fucose:H+ symporter permease
fig|6666666.1078954.peg.1233	T3S	domain-containing protein
fig|6666666.1078954.peg.1251	T3S	CD225/dispanin family protein
fig|6666666.1078954.peg.1314	T3S	4-hydroxyphenylpyruvate dioxygenase
fig|6666666.1078954.peg.1318	T3S	oligopeptide:H+ symporter
fig|6666666.1078954.peg.1319	T3S	Tryptophan 2,3-dioxygenase
fig|6666666.1078954.peg.1356	T3S	XopA/Hpa1 family type III secretion system protein
fig|6666666.1078954.peg.1365	T3S	type III secretion protein HrpB2
fig|6666666.1078954.peg.1373	T3S	4-hydroxyphenylacetate catabolism regulator HpaA
X. citri pv. *viticola* CCRM116		
Protein	Prediction	Description
fig|6666666.1078955.peg.2736	T3S	peptidoglycan-binding protein
fig|6666666.1078955.peg.1117	T3S	cation diffusion facilitator family transporter
fig|6666666.1078955.peg.1213	T3S	efflux RND transporter periplasmic adaptor subunit
fig|6666666.1078955.peg.1214	T3S	DHA2 family efflux MFS transporter permease subunit
fig|6666666.1078955.peg.1219	T3S	hypothetical protein
fig|6666666.1078955.peg.1223	T3S	M3 family metallopeptidase
fig|6666666.1078955.peg.124	T3S	oligopeptide:H+ symporter
fig|6666666.1078955.peg.128	T3S	4-hydroxyphenylpyruvate dioxygenase
fig|6666666.1078955.peg.1336	T3S	L-fucose:H+ symporter permease
fig|6666666.1078955.peg.1378	T3S	ArdC-like ssDNA-binding domain-containing protein

X. citri pv. *viticola* CCRM124		
Protein	Prediction	Description
fig|6666666.1078956.peg.2924	T3S	peptidoglycan-binding protein
fig|6666666.1078956.peg.117	T3S	L-fucose:H+ symporter permease
fig|6666666.1078956.peg.1193	T3S	lysophosphatidylcholine acyltransferase
fig|6666666.1078956.peg.1223	T3S	hypothetical protein
fig|6666666.1078956.peg.1298	T3S	helix-turn-helix domain-containing protein
fig|6666666.1078956.peg.1374	T3S	MFS transporter
fig|6666666.1078956.peg.1420	T3S	NYN domain-containing protein
fig|6666666.1078956.peg.1421	T3S	hypothetical protein
fig|6666666.1078956.peg.1468	T3S	ArdC-like ssDNA-binding domain-containing protein
fig|6666666.1078956.peg.1483	T3S	hypothetical protein
X. citri pv. *viticola* CCRM154		
Protein	Prediction	Description
fig|6666666.1076499.peg.1895	T3S	peptidoglycan-binding protein
fig|6666666.1076499.peg.1116	T3S	cation diffusion facilitator family transporter
fig|6666666.1076499.peg.1213	T3S	efflux RND transporter periplasmic adaptor subunit
fig|6666666.1076499.peg.1214	T3S	DHA2 family efflux MFS transporter permease subunit
fig|6666666.1076499.peg.1219	T3S	hypothetical protein
fig|6666666.1076499.peg.1223	T3S	M3 family metallopeptidase
fig|6666666.1076499.peg.123	T3S	oligopeptide:H+ symporter
fig|6666666.1076499.peg.127	T3S	4-hydroxyphenylpyruvate dioxygenase
fig|6666666.1076499.peg.1336	T3S	L-fucose:H+ symporter permease
fig|6666666.1076499.peg.1439	T3S	TonB-like protein
X. citri pv. *viticola* CCRM214		
Protein	Prediction	Description
fig|6666666.1076491.peg.2046	T3S	peptidoglycan-binding protein
fig|6666666.1076491.peg.119	T3S	L-fucose:H+ symporter permease
fig|6666666.1076491.peg.1248	T3S	DUF3426 domain-containing protein
fig|6666666.1076491.peg.1266	T3S	CD225/dispanin family protein
fig|6666666.1076491.peg.1329	T3S	4-hydroxyphenylpyruvate dioxygenase
fig|6666666.1076491.peg.1333	T3S	oligopeptide:H+ symporter
fig|6666666.1076491.peg.1372	T3S	XopA/Hpa1 family type III secretion system protein
fig|6666666.1076491.peg.1381	T3S	type III secretion protein HrpB2
fig|6666666.1076491.peg.1389	T3S	4-hydroxyphenylacetate catabolism regulator HpaA
fig|6666666.1076491.peg.1394	T3S	hypothetical protein
X. citri pv. *viticola* CCRM230		
Protein	Prediction	Description
fig|6666666.1076500.peg.1803	T3S	peptidoglycan-binding protein
fig|6666666.1076500.peg.1111	T3S	cation diffusion facilitator family transporter
fig|6666666.1076500.peg.120	T3S	oligopeptide:H+ symporter
fig|6666666.1076500.peg.1207	T3S	efflux RND transporter periplasmic adaptor subunit
fig|6666666.1076500.peg.1208	T3S	DHA2 family efflux MFS transporter permease

		subunit
fig|6666666.1076500.peg.1213	T3S	hypothetical protein
fig|6666666.1076500.peg.1217	T3S	M3 family metallopeptidase
fig|6666666.1076500.peg.124	T3S	4-hydroxyphenylpyruvate dioxygenase
fig|6666666.1076500.peg.1331	T3S	L-fucose:H+ symporter permease
fig|6666666.1076500.peg.1432	T3S	TonB-like protein
X. citri pv. *viticola* CCRM234		
Protein	Prediction	Description
fig|6666666.1076503.peg.1791	T3S	peptidoglycan-binding protein
fig|6666666.1076503.peg.1113	T3S	cation diffusion facilitator family transporter
fig|6666666.1076503.peg.1209	T3S	efflux RND transporter periplasmic adaptor subunit
fig|6666666.1076503.peg.1210	T3S	DHA2 family efflux MFS transporter permease subunit
fig|6666666.1076503.peg.1215	T3S	hypothetical protein
fig|6666666.1076503.peg.1219	T3S	M3 family metallopeptidase
fig|6666666.1076503.peg.124	T3S	oligopeptide:H+ symporter
fig|6666666.1076503.peg.128	T3S	4-hydroxyphenylpyruvate dioxygenase
fig|6666666.1076503.peg.1332	T3S	L-fucose:H+ symporter permease
fig|6666666.1076503.peg.1434	T3S	TonB-like protein
